# Revisiting Wölfflin in the Age of AI: A Study of Classical and Baroque Composition in Generative Models

**DOI:** 10.3390/jimaging11050128

**Published:** 2025-04-22

**Authors:** Adrien Deliege, Maria Giulia Dondero, Enzo D’Armenio

**Affiliations:** 1 Department of Romance Languages and Literatures, Faculty of Philosophy and Letters, University of Liège, 4000 Liège, Belgium; mariagiulia.dondero@uliege.be; 2 Department of Electrical Engineering and Computer Science, Montefiore Institute, Faculty of Applied Sciences, University of Liège, 4000 Liège, Belgium; 3 F.R.S.-FNRS, Rue d’Egmont 5, 1000 Bruxelles, Belgium; 4 UFR Sciences Humaines et Sociales, Université de Lorraine, 57000 Metz, France; enzo.d-armenio@univ-lorraine.fr

**Keywords:** generative AI, Wölfflin categories, visual semiotics, Classical painting, Baroque painting, text-to-image models, art history, DALL•E, Midjourney

## Abstract

This study explores how contemporary text-to-image models interpret and generate Classical and Baroque styles under Wölfflin’s framework—two categories that are atemporal and transversal across media. Our goal is to see whether generative AI can replicate the nuanced stylistic cues that art historians attribute to them. We prompted two popular models (DALL•E and Midjourney) using explicit style labels (e.g., “baroque” and “classical”) as well as more implicit cues (e.g., “dynamic”, “static”, or reworked Wölfflin descriptors). We then collected expert ratings and conducted broader qualitative reviews to assess how each output aligned with Wölfflin’s characteristics. Our findings suggest that the term “baroque” usually evokes features recognizable in typically historical Baroque artworks, while “classical” often yields less distinct results, particularly when a specified genre (portrait, still life) imposes a centered, closed-form composition. Removing explicit style labels may produce highly abstract images, revealing that Wölfflin’s descriptors alone may be insufficient to convey Classical or Baroque styles efficiently. Interestingly, the term “dynamic” gives rather chaotic images, yet this chaos is somehow ordered, centered, and has an almost Classical feel. Altogether, these observations highlight the complexity of bridging canonical stylistic frameworks and contemporary AI training biases, underscoring the need to update or refine Wölfflin’s atemporal categories to accommodate how generative models—and modern visual culture—reinterpret Classical and Baroque.

## 1. Introduction

Still images have long served as a primary medium for communicating visual ideas and emotions, from the earliest cave paintings to contemporary digital artwork. Even if their visual substrate lacks actual motion, certain images possess a powerful sense of “movement” or *dynamism*—an impression conveyed purely through compositional elements such as line, color, shape, and light, which we will call *compositional dynamism* and study in more detail. While art historians have historically debated the roots and mechanisms behind this sensation of movement, the recent emergence of generative text-to-image models introduces a new perspective as follows: machines that can produce novel, often highly expressive visuals based on text prompts. As these models become increasingly prevalent in both commercial and creative spheres, it is essential to understand whether and how they encode aesthetic constructs that humans have developed over centuries.

From a visual semiotic and art historical perspective, whether static images can truly convey motion has always been a question of interest. Traditionally, painting and sculpture have been cast as “arts of space”, seemingly unable to capture successive events central to “arts of time” like poetry or music [1,2]. However, scholars such as Groupe μ and Petitot argue that still images *simulate* temporality by relying on compositional strategies—diagonals, overlapping forms, or blurred outlines—that imply movement [3,4,5]. These insights underscore the tension between the material fixity of a two-dimensional plane and the viewer’s perception of energy or duration, suggesting that even static artworks can gesture toward narrative or process-based dynamism.

One of the foundational theories of visual style and composition is derived from Heinrich Wölfflin’s *Principles of Art History* [6,7], which outlines five pairs of opposing categories to distinguish the so-called *classical* mode of depiction from the *Baroque*. Wölfflin’s Baroque category, in particular, is often associated with diagonal lines, dramatic lighting, and intertwined shapes that create a heightened sense of movement or energy. His framework has proven to be enduring in art historical discussions, influencing how scholars interpret painting, sculpture, and architecture. However, Wölfflin’s categories originated in early twentieth-century Europe, well before the advent of digital art and artificial intelligence, prompting a debate about the universality and adaptability of his insights when facing new media and new cultural contexts.

Generative text-to-image models, such as those that leverage diffusion or generative adversarial network architectures, draw from large-scale image databases that span multiple epochs, styles, and cultural sources. This situation raises the following key questions: *To what extent do these AI models internalize compositional strategies that we might label as “classical” or “baroque”? Can they replicate the sense of dynamism that art historians identify in a Bernini sculpture or a Caravaggio painting? And do they inadvertently introduce new forms of dynamism beyond what Wölfflin could have foreseen?* While early demonstrations of generative models have been both celebrated and critiqued, systematic research on how these models portray concepts like movement and compositional tension remains limited.

**Contributions.** This study, therefore, aims to bridge Classical art historical theory with contemporary computational tools. First, we propose an operational definition of “dynamism” in still images, rooted in Wölfflin’s five oppositions. Next, we explore how text-to-image models respond to prompts invoking either “classical” or “baroque” motifs through expert evaluations performed blindly and then holistically. Finally, we examine how well these generative systems capture Wölfflin’s nuanced compositional differences when faced with varying levels of stylistic specificity in the prompt (including the absence of style labels or the introduction of certain genres). By combining historical, theoretical, semiotic, and computational approaches, we hope to offer fresh insights into the longevity of Wölfflin’s principles, as well as to shed light on the evolving landscape of AI-driven image generation.

## 2. Background and Related Work

### 2.1. Visual Semiotics and the Still/Moving Image Debate

Art philosophy and aesthetics have traditionally positioned painting and sculpture as *arts of space*, grounded in the juxtaposition of elements on a flat or sculptural substrate [1,2]. By contrast, *arts of time*—such as poetry and music—are described as unfolding through successive intervals and events. This conceptual divide raises the following fundamental question: *how might a static artwork convey any real sense of temporality or motion*?

While some scholars have long maintained that images cannot truly replicate the sequential dimension of time, some visual semioticians (and rare art philosophers, such as Pinotti [1]) marked a significant conceptual change by positing that still artworks *simulate* movement through compositional strategies. Groupe μ [3], for instance, highlights how images can evoke motion through implied actions or gestures, suggesting that objects like a rolling ball or a cutting knife carry latent dynamism into a scene. We will call this dynamism *semantic dynamism*, that is, dynamism depicting an action, where motion is implied by the semantic nature of the entities represented. Petitot likewise explores how morphological cues—curves, diagonals, or certain chromatic contrasts—can signal underlying energy and tension [4,5]. In effect, his theory emphasizes the “plane of expression” (line, form, color) rather than strictly figurative or narrative components when examining motion in still images [3]. We will call this dynamism *compositional dynamism*, that is, dynamism created by compositional techniques. Dondero argues that even seemingly inert subjects (e.g., portraits) can be structured in ways that invite the viewer to perceive temporal processes or tensions, thereby transcending the strict boundaries of an art of space [2,8,9].

Such semiotic perspectives complicate the traditional assumption that static images cannot convey duration. They also highlight the importance of subtle plastic devices—such as overlapping forms, blurred contours, or gestural traces—that prompt the viewer to imagine a time-based sequence. In doing so, the still image becomes an arena where dynamism can be *inferred* rather than literally depicted. We position our paper in the study of *compositional dynamism*, which is tied to Wölfflin’s seminal work on the analysis of artworks, and which is also the most nuanced and challenging type of dynamism that a text-to-image model can produce, as not directly linked to the content of the images.

### 2.2. Wölfflin’s Classical/Baroque Framework

Among the most influential art historical theories on visual style is Heinrich Wölfflin’s *Principles of Art History* [6,7]. He outlined five pairs of opposing categories through which he distinguished the “classical” mode of depiction from the “baroque”. These well-known pairs are as follows:

- **Linear vs. Painterly (Line vs. Mass):** Classical works emphasize clear outlines and contours, whereas Baroque works merge or dissolve contours to create a sense of mass or flow.
- **Planar vs. Recessional:** Classical compositions arrange elements along distinct frontal planes, while Baroque compositions unify the spatial field, often using diagonals that lead the viewer’s gaze into depth.
- **Closed vs. Open form:** In Classical works, the composition is typically self-contained, aligning neatly with the frame. By contrast, Baroque images often “cut” into the larger world, creating the impression of an expansive scene that continues beyond the canvas.
- **Multiplicity vs. Unity:** Classical art features clearly delineated, independent elements, whereas Baroque art fuses elements into an indivisible whole.
- Clearness vs. Unclearness: Classical lighting clarifies shape and form distinctly, whereas Baroque lighting, often intense and with high contrast, creates ambiguity, merging form and shadow for heightened emotional effect.

**Figure 1:**
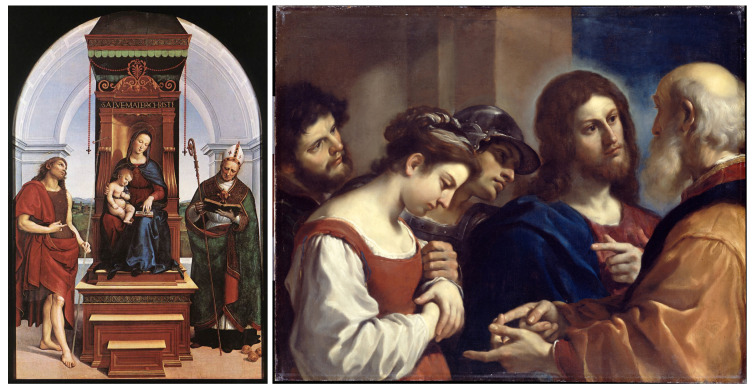
**Left**: a typical Classical painting of Raphaël, *The Ansidei Madonna*; **right**: a typical Baroque painting of Guercino, *The Woman taken in Adultery*.

Typical paintings exemplifying these five Classical vs. Baroque oppositions are shown in Figure 1, as detailed in [10].

Wölfflin argued that while these five pairs emerged historically during the Renaissance and Baroque periods, they also serve as *timeless* or *trans-medial* categories applicable to diverse artworks and styles [6,7]. Notably, his description of Baroque dynamism—anchored in diagonal lines, intense light/shadow contrasts, and swirling masses—has particular resonance for scholars exploring the evocation of dynamism in still images. At the same time, critics point out that Wölfflin’s categories derived primarily from European art of the sixteenth and seventeenth centuries, and may not seamlessly map onto global or modernist creations [11]. As such, researchers continue to debate whether these dualities are truly universal or instead reflect a specific cultural and historical perspective. For instance, Goude and Derefeldt [12] propose an empirical test of Wölfflin’s original system by asking both trained art-history students and untrained psychology students to rate Renaissance-to-Baroque paintings on his five pairs of concepts. Their findings show that even naïve observers can apply most of Wölfflin’s categories consistently and in line with historical expectations, although some concepts (like clearness vs. unclearness) prove less robust [12]. Unlike their study of authentic Renaissance and Baroque artworks, we explore how generative AI interprets or fails to interpret Wölfflin’s distinctions in purely synthetic images. Besides, on a general note, given the importance of Wölfflin’s work in the study of visual arts, it makes sense for us to take it as reference for our comparative study.

### 2.3. Art-Historical Approaches to Movement and Dynamism

Beyond Wölfflin, numerous art historical precedents shed light on how artists have coped with the representation of motion in static media. During the Renaissance, painters like Sassetta sometimes placed multiple narrative moments within a single frame, effectively creating a temporal sequence on a single canvas [1]. In the nineteenth century, chronophotography by Eadweard Muybridge or Etienne-Jules Marey broke down movement into overlapping, successive snapshots, producing an early form of motion study on a two-dimensional plane [13].

By the early twentieth century, Italian futurists (Marinetti, Boccioni, Balla, and others) explicitly championed speed and energy, employing repeated patterns or blurred outlines to suggest velocity [14]. Kandinsky and Klee, though less literal in their depictions, advanced theories of abstraction wherein lines, points, and color fields could convey a dynamic inner rhythm [15,16]. Jackson Pollock famously took this logic further by transferring the physical gestures of his painting process directly onto the canvas, making the viewer acutely aware of the artist’s bodily motion [13].

In many of these cases, one can discern partial parallels to Wölfflin’s Baroque dimension. Diagonal compositions, swirling forms, or dramatic color contrasts play a key role in “activating” a static image. This continuity suggests that while futurists or abstract expressionists were not directly engaging with Wölfflin’s framework, their formal strategies to evoke motion share a core vocabulary with the Baroque momentum toward dynamism [17].

More recently, Baroque-inspired visual strategies have seen renewed interest in modern film and photography, where intense lighting, diagonal framing, and sweeping camera movements are deliberately used to evoke a sense of heightened drama. Filmmakers often harness strong chiaroscuro, swirling camera angles, and abrupt changes in scale to instill motion even in static scenes—thereby recapturing the same compositional tension that Wölfflin attributed to Baroque art. In photography, tightly cropped or partially obscured elements can similarly convey the impression of an expansive, continuous world beyond the frame, further echoing Baroque’s open form. Such revivals underscore Wölfflin’s lingering influence; his Classical/Baroque lens continues to illuminate how contemporary visual culture orchestrates the feeling of dynamism in still images. On our side, we limit our study to the image domain, leaving subsequent analyses for future works.

### 2.4. Computational Analyses of Artwork and Style

With the digitization of large museum collections, computer vision methods have become increasingly relevant to art historical studies [18,19]. Early computational approaches focused on basic color histograms or texture descriptors to cluster or compare artworks [19,20], but recent advances incorporate more sophisticated methods for style recognition, pose estimation, and object detection in paintings [21,22,23,24,25,26,27].

Researchers have also attempted to quantify visual dynamism in paintings. For instance, [28] used edge orientations to measure the prevalence of diagonals versus vertical or horizontal lines, linking diagonal dominance to a heightened sense of motion. Alongside these traditional feature-based approaches, deep learning has enabled tasks such as painting classification, retrieval, and even “visual question answering” for art historical questions [29,30,31,32].

More closely related to our work, Jha et al. [33] introduce “Wölfflin Affective Generative Analysis” to investigate how generative adversarial networks compare to authentic historical artworks in how non-expert audiences emotionally respond to those generated images. Their focus lies in contrasting real vs. machine-made art at scale, examining correlations between Wölfflin’s categories and human-reported affect. In contrast, our study focuses on prompting strategies and analyses of powerful text-to-image diffusion models for Baroque or Classical styles and examines their compositional outputs through expert-based evaluations, without considering the emotional aspect.

Yet, to the best of our knowledge, no study directly addresses how compositional factors as derived by Wölfflin might be automatically identified and measured in the context of dynamism. While we do not aim to automate that process, we are still likely the first to study such factors through the lens of modern image generators.

### 2.5. Bridging Canonical Theory and Generative AI

Recent developments in *generative* models—particularly diffusion-based approaches and generative adversarial networks—provide new avenues for both creating and studying art. Systems such as DALL•E (https://openai.com/index/dall-e-3/ (accessed on 18 February 2025)), Midjourney (https://www.midjourney.com/home (accessed on 18 February 2025)), and Stable Diffusion (https://stability.ai/stable-image (accessed on 18 February 2025)) have shown remarkable flexibility in producing stylistically diverse images from text prompts, ranging from photorealism to impressionist or surreal styles [34,35,36]. While some efforts have explored how these models can replicate art historical styles or seamlessly combine them (e.g., “Van Gogh + pop art”), few have investigated whether generative models internalize deeper compositional principles—like those identified by Wölfflin—or produce “dynamism” in alignment with semiotic debates about the still image.

This is what we study in this paper. That gap points to a pressing research need; an examination of whether AI’s latent space captures and reproduces common Baroque triggers for dynamism (e.g., swirling diagonals, dramatic lighting), or whether it instead innovates new formal strategies—akin, perhaps, to the micro-repetitions of small strokes typical of futurism that Wölfflin could not have foreseen. In bridging canonical art historical theory with state-of-the-art generative capabilities, we can test the limits and adaptability of both. Ultimately, this study aims to build on semiotic and art historical insights about the still image, using computational methods to probe how a new class of algorithms reimagines “dynamism” in the static frame.

## 3. Method

In this section, we describe our systematic exploration of how text-to-image models interpret Classical and Baroque stylistic prompts, as well as prompts concerning dynamism versus stasis. We employed two generative models, DALL•E 3 and Midjourney v6, and designed five distinct experiments (Section 3.2.1, Section 3.2.2, Section 3.2.3, Section 3.2.4 and Section 3.2.5). Two experts in visual semiotics and art history served as our evaluators, rating selected images using a Wölfflin-inspired scheme (Section 3.3).

### 3.1. Models and Sampling Procedure

We used two popular generative models as follows: DALL•E 3 accessed via the OpenAI API with default settings (creative or “prompt-based” mode), and Midjourney v6 running in its standard mode with a random seed for each image. Unless otherwise specified, we generated 10 images per prompt per model. Each image was produced with a unique random seed to ensure variability. We stored all generated images with anonymized filenames (omitting any mention of the model or prompt) for later evaluation.

### 3.2. Experimental Conditions

We organized our experiments into five main sets of prompts, summarized below.

#### 3.2.1. Experiment 1: Neutral vs. Classical vs. Baroque

This experiment aimed to observe how the models interpret generic style words (*Classical* and *Baroque*), compared to a neutral baseline, through three prompts: “*A painting*” (neutral), “*A classical painting*”, “*A baroque painting*”. For each of the three prompts, we generated 10 images in DALL•E and 10 images in Midjourney, yielding 60 images total. The objective of this first experiment is to gauge how each model spontaneously interprets the terms “classical” and “baroque” when no other compositional constraints are applied. By comparing outputs from these prompts, we want to observe the models’ baseline stylistic biases, i.e., whether the word “baroque” triggers diagonal compositions, swirling forms, or heightened chiaroscuro, and whether “classical” yields balanced, orderly imagery. The neutral prompt provides a control reference, indicating the default or most common painting style each model tends to generate in the absence of explicit stylistic instructions.

#### 3.2.2. Experiment 2: Adding a Genre (Portrait)

To test whether genre constraints overshadow stylistic prompts, we repeated the procedure of Experiment 1 but fixed the subject as “*a portrait*”, through the following three prompts: “*A painting of a portrait*” (neutral), “*A classical painting of a portrait*”, “*A baroque painting of a portrait*”. Similarly, 10 images per prompt per model were produced, for a total of 60 images. While Experiment 1 revealed how models treat “classical” and “baroque” in a generic sense, this second experiment aims to determine whether those stylistic cues remain consistent once a specific genre is introduced. Portraits, in particular, come with strong conventions for subject placement, facial detail, and lighting, which may overshadow or reinforce the stylistic prompts. By comparing these prompts, we can assess the relative influence of genre constraints versus stylistic terms. This comparison clarifies whether adding a subject type amplifies or diminishes the compositional cues associated with Classical or Baroque characteristics.

Note on other genres: We generated similar prompts for “*A painting of a still life*” and “*A painting of a landscape*”, but we did not conduct a formal rating session. Qualitative inspection suggested outcomes analogous to those for portraits, hence we omitted a costly evaluator pass for these additional genres.

#### 3.2.3. Experiment 3: Wölfflin Descriptors Without Style Labels

In the third experiment, we tested whether explicitly describing Classical or Baroque compositional principles (drawn from Wölfflin), *without using the words “classical” or “baroque”*, might yield the intended style. For the Classical theme, the prompt was “*A painting emphasizing clear, well-defined contours that precisely separate elements, ordered spatial layers arranged in distinct frontal planes, a self-contained and balanced composition neatly enclosed within the frame, clearly separated elements with independent significance that maintain structured parts, and uniform, evenly distributed lighting that ensures all elements are clearly visible with minimal shadow contrast*”. For the Baroque style, the prompt was “*A painting emphasizing blurred, merged contours that create a sense of mass of elements, unified space with strong diagonals and dramatic recession into depth, an unbalanced composition that extends beyond the frame, visually interwoven elements that blend together into a cohesive whole, and dramatic contrasts of light and shadow that obscure some elements*”. We generated 10 images per descriptive prompt per model, for a total of 40 images. Having observed in Experiments 1 and 2 that simply naming styles (“classical” and “baroque”) does not always yield clear compositional differences, Experiment 3 focuses on whether *explicit Wölfflinian descriptors* can more directly induce Classical or Baroque aesthetics. By providing the model with detailed instructions but deliberately *omitting* the words “classical” and “baroque”, we aim to test if the model can replicate these compositional principles purely from descriptive language.

#### 3.2.4. Experiment 4: Dynamic vs. Static

Recognizing that Wölfflin’s *Baroque* is often associated with a sense of movement or dynamism, we contrasted prompts using the words “*dynamic*” and “*static*”, as follows: “*A dynamic painting*”, “*A static painting*”. Again, 10 images per prompt per model weer generated, totaling 40 images. Since the concept of dynamism is central to Wölfflin’s notion of the Baroque, we wanted to see whether the simpler terms “dynamic” and “static” would elicit any clearer distinction in the models. By comparing these prompts, we examine whether the models automatically associate movement (and potential Baroque-like cues) with the former, and stillness (possibly linked to Classical ideals) with the latter.

#### 3.2.5. Experiment 5: Dynamic vs. Static with a Genre (Portrait)

Finally, we replicated Experiment 4’s distinction between *dynamic* and *static* painting, but introduced the keyword “*of a portrait*” to observe genre effects through the following prompts: “*A dynamic painting of a portrait*” and “*A static painting of a portrait*”. Again, we generated 10 images per prompt per model, resulting in 40 images. Building on the logic of Experiment 4, this final experiment examines whether specifying a subject type (here, “*a portrait*”) alters the models’ interpretation of “dynamic” versus “static”. As with Classical and Baroque in Experiment 2, we anticipate that traditional portrait conventions (e.g., centering a figure, with an equilibrium between the figure and the background, and the figure being compact, i.e., not dispersed in the background through blur) may constrain how far a model will push dynamism or stillness.

### 3.3. Rating Protocol and Blind Evaluation

*Expert evaluators.* Two experienced visual semioticians served as our evaluators. For Experiments 1 and 2, each of the 60 images (per experiment) was rated on a set of Likert scales from 1 (strongly Classical) to 5 (strongly Baroque). These scales included the following: (a) *General Feeling:* overall Classical vs. Baroque impression; (b) *Linear vs. Painterly*; (c) *Planar vs. Recessional*; (d) *Closed vs. Open Form*; (e) *Multiplicity vs. Unity*; (f) *Clearness vs. Unclearness*.

**Figure 2:**
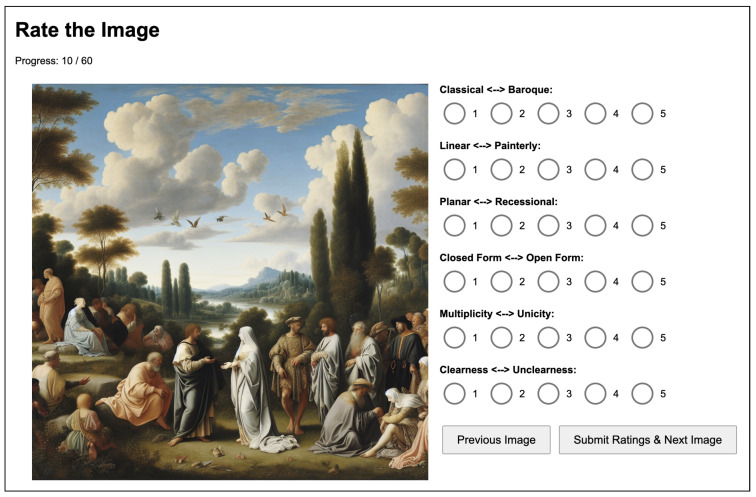
Wölfflin rating system based on the Likert scales used in our experiments, freely available at https://wolfflin-rating-app.azurewebsites.net/ (accessed on 18 February 2025).

*Anonymization and randomization.* For Experiments 1 and 2, we renamed each image with a random numeric ID and randomized their display order, ensuring the evaluator did not know which prompt or which model generated a particular image. Ratings were entered via a simple web interface, as shown in Figure 2, and then exported as a spreadsheet for analysis.

*Data analysis.* After the evaluation, we computed the average Likert ratings for each style/genre combination per model, enabling direct comparisons (e.g., *Baroque vs. Classical* vs. *neutral*; *portrait vs. no portrait*). Qualitative notes from the evaluators were also recorded, particularly for Experiments 3–5, to identify emergent themes (e.g., abstract shapes vs. figurative composition; presence vs. absence of swirling diagonals).

#### Post-Rating Review and Discussion

After completing all the blind, randomized evaluations described above, we asked the same experts to revisit each experiment’s full image set in a more holistic manner. This time, the prompts and model sources were revealed (e.g., which images belonged to “classical”, “baroque”, “dynamic”, or “static”), allowing the experts to see all generated outputs for a given experiment side by side. The purpose of this post-rating review was not to gather additional Likert scores, but to facilitate a broader discussion informed by complete visual context and the “ground truth” of how each image was produced. In practice, the experts gathered to observe and compare the images collectively, noting emergent patterns or discrepancies that had been less apparent during the earlier, blind evaluations. Their informal comments and observations offered a deeper understanding of the compositional trends in each prompt condition—insights that would be difficult to obtain from isolated, randomized image presentations alone. We recorded these expert discussions qualitatively, capturing how style cues might manifest more clearly (or become diluted) when images from different prompts were directly juxtaposed. This open-ended, holistic feedback then supported and enriched the quantitative analyses derived from the blind rating procedures.

## 4. Results and Discussion

We present and discuss the results of the five experiments described in Section 3. Then, we report the outcome of the general discussion between the experts about the generated images, as described in Section Section 3.3.

### 4.1. Analysis of Experiment 1 Results

**Figure 3:**
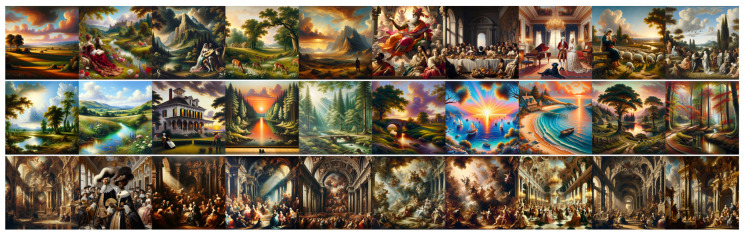
Images produced by DALL•E for Experiment 1. **Top line**: “*A classical painting*”; **middle line**: “*A painting*” (neutral); and **bottom line**: “*A baroque painting*”.

**Figure 4:**
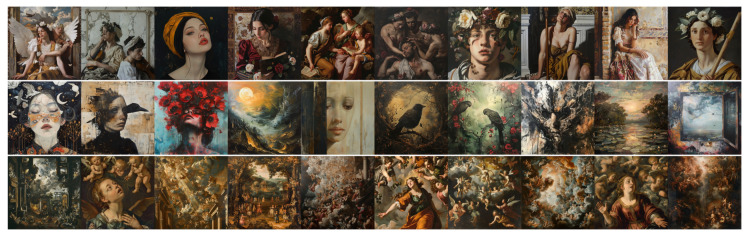
Images produced by Midjourney for Experiment 1. **Top line**: “*A classical painting*”; **middle line**: “*A painting*” (neutral); and **bottom line**: “*A baroque painting*”.

Figure 3 and Figure 4 show the images produced for this experiment, and Table 1 shows the average Likert scores from our two expert evaluators (E1 and E2) for these images, as detailed in Section 3.2.1. Each score ranges from 1 (strongly Classical) to 5 (strongly Baroque) and reflects a general feeling and the five Wölfflin dimensions as follows: (1) General Feeling; (2) Linear vs. Painterly; (3) Planar vs. Recessional; (4) Closed vs. Open Form; (5) Multiplicity vs. Unity; and (6) Clearness vs. Unclearness.

A first observation is that E1 perceives a marked contrast between Baroque and Classical prompts for both models, with DALL•E Baroque consistently scoring above 3.3 on all dimensions (notably 4.4 for clearness), with DALL•E Classical remaining below 2.7 (notably 1.7 for linear vs. painterly). A similar gap appears in Midjourney for E1, where Baroque prompts are above 4.0 in 4 out of 6 cases, indicating a strong sense of dynamism or drama, and Classical images tend to hover near 2.0. This suggests that, from E1’s perspective, both models can generate images that align with a Baroque or Classical aesthetic when prompted, with a relatively clear distinction between the two.

E2’s ratings paint a more complex picture. For DALL•E, E2 likewise distinguishes Baroque (3.0–3.6) from Classical (2.0–2.3), though the numerical gap is narrower than with E1. Meanwhile, Midjourney prompts show a surprising overlap. Classical outputs are often rated as high as 3.2–3.8, on par with or exceeding Baroque prompts. This outcome indicates greater ambiguity in how E2 interprets the compositional cues of Midjourney images, implying that the model’s “Classical” or “Baroque” results may incorporate traits (such as high contrast or merged forms) that this expert finds harder to categorize.

Comparing the Neutral condition across both experts reveals additional insights. For E1, DALL•E’s neutral images generally cluster around 2.3, barely differing from the Classical ratings, whereas Midjourney’s neutral outputs revolve around 3.2, with ratings always comprising the Classical and Baroque ratings, indicating a clear distinction between the three regimes. E2 sees DALL•E’s neutral images as even more Classical (scores as low as 1.4 for Planar vs. Recessional) than the Classical images, yet finds Midjourney’s neutral prompts frequently match or exceed Baroque-level ratings (e.g., around 3.8 in general feeling). These findings suggest that Midjourney’s default visual style, at least as perceived by E2, tends to be significantly more dramatic or dynamic than DALL•E’s, potentially overshadowing the explicit style instructions of “baroque” or “classical”.

In summary, both experts perceive DALL•E’s “classical” and “baroque” conditions as relatively well separated, reinforcing the notion that DALL•E applies different compositional cues for these two styles. At the same time, for DALL•E, both E1 and E2 note that “classical” and “neutral” prompts score quite similarly, suggesting that DALL•E’s default (neutral) output does not diverge much from a Classical baseline. In contrast, Midjourney presents a more varied picture. E1 sees distinct groupings among the Classical, Baroque, and neutral categories, indicating three recognizable stylistic modes. However, E2’s ratings reveal extensive overlap across these prompts, often assigning higher (i.e., more Baroque-like) scores overall. This discrepancy implies that Midjourney’s underlying aesthetic biases, combined with E2’s interpretative frame, blur the lines between Classical and Baroque or even neutral. As such, Midjourney images can appear significantly more dramatic or “baroque” by default, but the distinction from intentionally prompted Baroque outputs is less pronounced for E2. Taken together, these observations underscore the interplay between a model’s default stylistic tendencies and an evaluator’s experience, ultimately shaping how faithfully generative outputs reflect canonical notions of Classical and Baroque compositions.

### 4.2. Analysis of Experiment 2 Results

**Figure 5:**
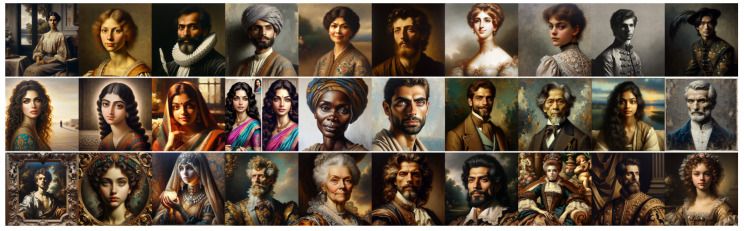
Images produced by DALL•E for Experiment 2. **Top line**: “*A classical painting of a portrait*”; **middle line**: “*A painting of a portrait*” (neutral); and **bottom line**: “*A baroque painting of a portrait*”.

**Figure 6:**
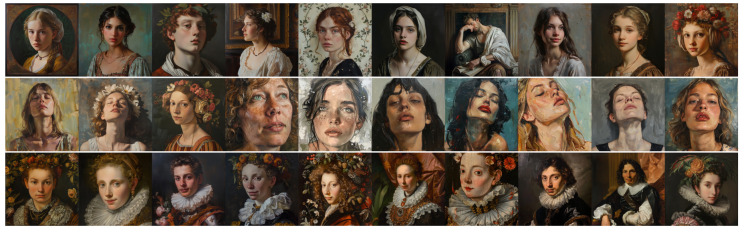
Images produced by Midjourney for Experiment 2. **Top line**: “*A classical painting of a portrait*”; **middle line**: “*A painting of a portrait*” (neutral); and **bottom line**: “*A baroque painting of a portrait*”.

Figure 5 and Figure 6 show the images produced for this experiment, and Table 2 reports the mean ratings from our two expert evaluators (E1 and E2) for these images, with the same conventions as previously.

Focusing on E1’s results, we observe that the difference between DALL•E’s Baroque and Classical portrait prompts is far less pronounced than in Experiment 1. In most Wölfflin categories, their scores cluster around 2.0–3.0, with the neutral condition again aligning closely with Classical rather than standing apart on its own. This indicates that once the prompt specifies a portrait, DALL•E no longer clearly separates Baroque from Classical as it did before. For Midjourney, E1’s ratings remain uniformly low across all three style conditions, with only one dimension (Clearness vs. Unclearness for Baroque) slightly exceeding 3.0. While Baroque scores tend to be marginally higher than Classical in most categories, the general feeling dimension is actually lower for Baroque than Classical, and neutral emerges with the highest overall impression. These unexpected outcomes highlight how constraining the model to a portrait subject may dilute or even invert the stylistic cues observed in Experiment 1, blurring Baroque and Classical distinctions for E1 in Midjourney’s outputs.

E2’s evaluations reinforce the tendency for DALL•E’s Baroque portraits to score marginally higher on Baroque cues than its Classical or neutral counterparts, but the numerical gaps are small. All scores but two lie within the 2.6–3.2 range, implying that DALL•E’s portrait-based outputs do not strongly diverge across Classical or Baroque conditions for this evaluator. Midjourney’s results for E2 reveal more striking anomalies as follows: “Classical”, with all scores above 3.0, is always rated higher than “Baroque”, suggesting that the “classical image” prompt may introduce dramatic forms that E2 finds quite Baroque in spirit. Moreover, the “Neutral” prompt occasionally yields equally elevated scores (3.8 in general feeling), underscoring how Midjourney’s aesthetic biases—and this evaluator’s interpretive framework—can blur the lines between what is nominally Classical, Baroque, or neither. The score difference between DALL•E and Midjourney is also reduced for E2 in this experiment, adding to the overall uncertainty pertaining to this experiment.

Taken together, these observations suggest that imposing the portrait genre on prompts further diminishes the style contrasts in certain cases, particularly for Midjourney. Although DALL•E continues to show a moderate Baroque–Classical gap for E1, both experts find that Classical and neutral conditions are often interchangeable for DALL•E, and E2 sees only subtle differences overall. Meanwhile, Midjourney exhibits strong variability as follows: E1 perceives a relatively even “flattening” of style across all three prompts, whereas E2 frequently considers Midjourney’s Classical and neutral outputs more dramatic than the Baroque ones. The net effect is that, once constrained by a portrait subject, neither model consistently translates “baroque” or “classical” into clearly distinct compositional treatments, reinforcing the idea that recognizable portrait conventions may overshadow the intended historical style cues.

In order to generalize these observations beyond the sole “portrait” genre, we conducted the same experiment with “landscapes” and “still lifes”, which are two other main topics in art history. The images are provided in Appendix A. They were not rated by our experts because, by quickly looking at them, they concluded that the same observations as for portraits applied; for both DALL•E and Midjourney, landscapes and still lifes are centered, classically composed with regular closed forms contained within the frame, excepted for the lighting which is often Baroque-like. We therefore focused our efforts on the next set of experiments.

### 4.3. Analysis of Experiment 3 Results

**Figure 7:**
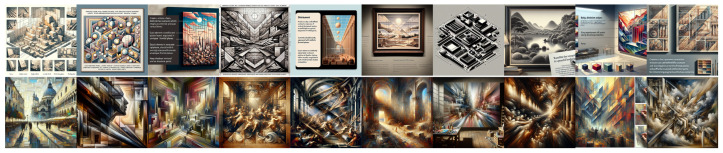
Images produced by DALL•E for Experiment 3. **Top line**: using a long descriptive prompt of Classical characteristics; **bottom line**: using a long descriptive prompt of Baroque characteristics.

**Figure 8:**
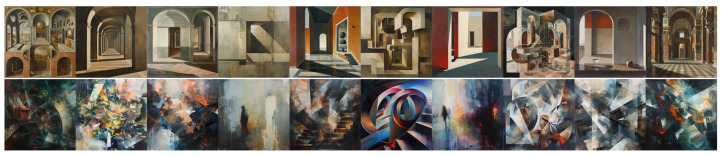
Images produced by Midjourney for Experiment 3. **Top line**: using a long descriptive prompt of Classical characteristics; **bottom line**: using a long descriptive prompt of Baroque characteristics.

Figure 7 and Figure 8 show the images produced for this experiment, and Table 3 reports the mean ratings from our two expert evaluators (E1 and E2) for these images, with the same conventions as previously.

Focusing first on E1, DALL•E Baroque tends to exceed DALL•E Classical by roughly one to two points in all categories, clearly separating the two styles. This pattern is also observed with Midjourney, whose Baroque ratings approach or exceed 3.0 in all columns, implying that Midjourney amplifies the compositional cues E1 associates with Baroque. Both models yield relatively low or moderate values under the “classical” label, with most dimensions falling below 2.5 for E1.

E2 draws attention to a different elements. DALL•E Baroque (3.6–3.9 across most columns) remains slightly higher than DALL•E Classical (2.3–3.5), but the gap narrows in dimensions like Planar vs. Recessional or Closed vs. Open Form. Meanwhile, Midjourney Baroque still draws the highest scores (3.7–4.0), highlighting its robust Baroque impression. Curiously, though, “MidJ Classical” reaches 3.0 or more in several columns (Linear vs. Painterly, Clearness vs. Unclearness), indicating that E2 finds elements of strong contrast or merged forms even under the “classical-oriented” label.

Overall, the data suggest that both experts consistently rate “baroque-oriented” prompts higher than “classical-oriented” prompts in each model, with Midjourney often producing especially dramatic outputs that rely more strongly on Baroque traits. However, E2’s ratings reveal smaller Baroque–Classical gaps in DALL•E and relatively elevated scores for “Midjourney Classical”, hinting at underlying differences in how each expert interprets compositional cues. These observations reinforce previous findings that while generative models do respond to Baroque vs. Classical prompts, the resulting stylistic separation remains imperfect and often depends on the evaluator’s experience. They also show that, despite the more "abstract" nature of the images generated, Wölfflin’s characteristics when explicitly prompted can still be transposed by the generative models in a distinctive way, albeit they might be less relevant in particular cases.

### 4.4. Analysis of Experiment 4 Results

**Figure 9:**
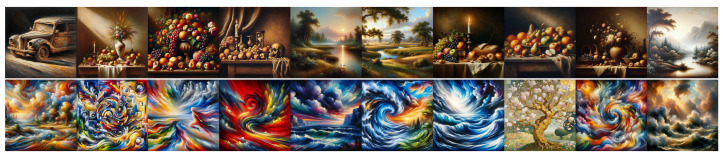
Images produced by DALL•E for Experiment 4. **Top line**: “*A static painting*”; **bottom line**: “*A dynamic painting*”.

**Figure 10:**
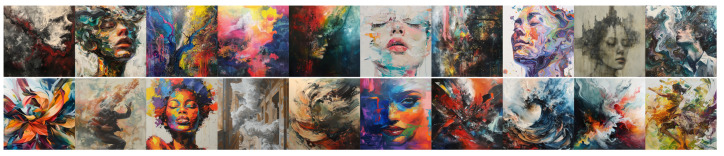
Images produced by Midjourney for Experiment 4. **Top line**: “*A static painting*”; **bottom line**: “*A dynamic painting*”.

Figure 9 and Figure 10 show the images produced for this experiment, and Table 4 reports the mean ratings from our two expert evaluators (E1 and E2) for these images, with the same conventions as previously.

For E1, DALL•E shows a noticeable gap between dynamic (2.7–3.4 ratings) and static (1.7–2.4), implying that “dynamic” produces images perceived as moderately more Baroque in Wölfflin dimensions, except for the clearness. However, Midjourney’s dynamic and static prompts do not differ significantly for E1, with nearly identical ratings in all categories. Importantly, this suggests that Midjourney’s outputs remain relatively similar regardless of whether the prompt requests a dynamic or static painting. To some extent, this aligns with the observations of D’Armenio et al. [37], who note that Midjourney consistently aims to beautify its images based on an internalized and stereotyped notion of what constitutes a painting with an aestheticizing ambition.

E2 perceives an even stronger distinction between dynamic and static in DALL•E, assigning scores above 3.0 for most categories of DALL•E dynamic, whereas DALL•E static hovers largely below 2.5, excepted again for clearness. This pattern indicates that the “dynamic” instruction leads DALL•E to generate paintings that E2 interprets as distinctly more Baroque. By contrast, Midjourney again appears less sensitive to these instructions for E2, with both dynamic and static prompts rated at or near 4.0 on several dimensions, indicating a consistently high Baroque-like feel, no matter whether the prompt specifies motion or stillness.

A notable point for both experts is that DALL•E’s dynamic prompts yield comparatively low clearness scores. Meanwhile, Midjourney’s clearness remains nearly the same for dynamic and static in both experts’ ratings, suggesting that Midjourney’s “dynamic” approach does not necessarily sacrifice clearness. Overall, these observations imply that DALL•E responds to “dynamic” vs. “static” with a stronger compositional shift than Midjourney does. E2, in particular, sees DALL•E dynamic as distinctly more Baroque-leaning than DALL•E static, whereas Midjourney’s outputs remain highly Baroque-like in both conditions, limiting the impact of these prompts.

**Importantly**, the experts also noted that most images were mostly “abstract”, and found it challenging to apply Wölfflin categories in this setting. Notably, they reported that this demanded a strong focus because it is unusual to rate the Baroqueness of abstract images, although these benefit as well from this analysis. They also underlined the idea that Wölfflin categories, while relevant for Classical vs. Baroque art and a large portion of art history, require a costly mental effort to be transposed to more recent (especially abstract) compositions. This observation does not only apply to images generated by AI models but also to several artistic movements of the last century. They suggested that novel categories might need to be proposed to refine the task of analyzing paintings or images in modern contexts. This need is reinforced by experiments such as the one presented above, as generative AI models are easily able to produce images that defy existing categories, while drawing inspiration from existing human creations. The possibility of generating easily many of them helps realizing this important yet possibly overlooked feat.

In addition, when examining Table 1 alongside Table 4, the magnitude and consistency of stylistic shifts differ across models and experts. In Experiment 1, *Baroque* vs. *Classical* prompts can yield relatively large gaps (e.g., DALL•E for E1 moves from 1.8 to 4.1 in General Feeling), whereas in Experiment 4, *dynamic* vs. *static* generally show milder differences for the same expert–model pairing. For instance, DALL•E with E1 ranges only from 1.9 to 2.7 under static and dynamic prompts, a narrower spread than between Classical and Baroque. However, for E2, the situation can be the reverse; sometimes “dynamic vs. static” produces as large or larger gaps than “baroque vs. classical”. Midjourney also exhibits peculiar behavior; in Experiment 4 it may conflate Baroque and Classical, yet in Experiment 1 some conditions appear strongly “baroque” or "classical" by default. Overall, the pattern suggests that specifying “baroque” or “classical” often triggers greater compositional separation for certain model–expert pairs, but “dynamic vs. static” can at times equal or exceed that effect, depending on each evaluator’s interpretive framework and each model’s baseline generative style.

### 4.5. Analysis of Experiment 5 Results

**Figure 11:**
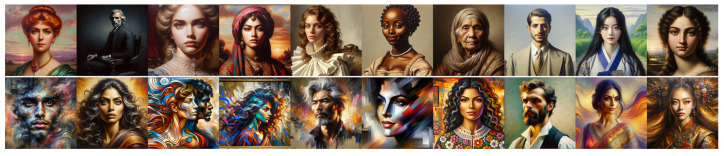
Images produced by DALL•E for Experiment 5. **Top line**: “*A static painting of a portrait*”; **bottom line**: “*A dynamic painting of a portrait*”.

**Figure 12:**
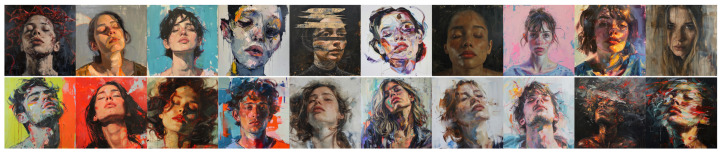
Images produced by Midjourney for Experiment 5. **Top line**: “*A static painting of a portrait*”; **bottom line**: “*A dynamic painting of a portrait*”.

Figure 11 and Figure 12 show the images produced for this experiment, and Table 5 reports the mean ratings from our two expert evaluators (E1 and E2) for these images, with the same conventions as previously.

Focusing on E1, DALL•E’s static portrait lands mostly in the 1.6–2.7 range, suggesting a Classical feel, while its dynamic portrait rises to 2.4–3.5 (especially for Closed vs. Open Form and Clearness vs. Unclearness), indicating a modest but consistent shift toward more Baroque-like traits. Midjourney exhibits smaller gaps between static and dynamic prompts, as in the previous experiment; for instance, the static portrait hovers around 2.1–2.9, whereas dynamic remains just slightly higher in General Feeling (3.1 vs. 2.4) but otherwise shows minimal differences in dimensions. This indicates that Midjourney again does make a significant difference between the prompts, even when the portrait genre is specified.

E2 sees a slightly sharper distinction for DALL•E, rating its static portrait below 2.0 in most columns (strongly Classical) but pushing the dynamic version nearer 3.0 for several dimensions. By contrast, Midjourney’s static and dynamic prompts yield similarly elevated scores (roughly 3.1–4.0), implying a generally “baroque” bent for *both* conditions—even “static” is perceived as relatively dramatic or merged. This high baseline for Midjourney’s portraits dilutes any compositional shift from “static” to “dynamic”, echoing prior observations that Midjourney’s default aesthetic often appears dynamic or high-contrast regardless of the prompt.

Overall, specifying “dynamic” versus “static” in a portrait context drives a clearer stylistic separation in DALL•E, whereas Midjourney remains fairly consistent—and fairly Baroque—across both prompts, particularly according to E2’s evaluations.

A direct comparison of Table 2 and Table 5 reveals that the relative impact of “dynamic vs. static” versus “classical vs. baroque” depends markedly on both the model and the expert, as already observed in the comparison of Experiments 1 and 4. For DALL•E, E2 observes a larger stylistic gap in Experiment 5 (dynamic–static) than in Experiment 2 (Classical–Baroque), while E1 perceives similar-sized distinctions in both experiments. In Midjourney, E1 sees a more pronounced difference for dynamic–static prompts than for Classical–Baroque, but E2 experiences the opposite, finding only minimal variation between Midjourney’s dynamic and static prompts, yet a stronger (albeit partially reversed) gap for Classical–Baroque. These contrasting outcomes suggest that neither static/dynamic nor Classical/Baroque categorization consistently dominates; rather, each model’s inherent style biases, combined with individual evaluators’ interpretive frames, shape how effectively a given prompt drives compositional change in portrait scenarios.

### 4.6. Expert Discussions After Holistic Viewing

Once the experts had completed their blind, randomized ratings for each experiment, we invited them to examine all images from a given prompt condition *en bloc*, with full knowledge of which prompt generated which image, as described in Section Section 3.3. This open review prompted a series of observations and reflections, which are summarized below.

*Genre constraints vs. Baroque/Classical cues.* When a specific genre (portrait, still life, or landscape) is specified, many outputs lean towards *Classical*, irrespective of nominally “open” framing. In Midjourney, for instance, figures or objects remain rigidly centered, impeding a genuinely open-form impression. Specifying the genre also imposes a characteristic compactness—a constant figure–ground relationship typical of portraiture, but transposed to other genres. Even when the prompt states “baroque”, only one Wölfflin trait (Unclearness) consistently appears; faces or vases in the center retain closed lines and shapes. By contrast, if we remove explicit mention of the genre and simply say “baroque”, the outputs become visibly more “baroque”, often with interlaced bodies—perhaps a stereotypical association for the AI. Conversely, if we omit the term “baroque” yet retain the reworked Wölfflin descriptors, the images skew toward abstract compositions. This suggests the model ties “baroque” strongly to figurative entanglements (as in historical seventeenth-century Baroque).

*Thematic vs. compositional tensions.* Multiple prompts show an intriguing divergence between theme and layout as follows: Baroque “themes” (swirling figures and rich ornamentation) might still adhere to a strictly Classical structure (e.g., a perfectly centered circle). Sometimes, images deemed “classical” in a broader sense earn Baroque-like ratings on clearness, perhaps due to dramatic lighting or high contrast. When we prompt “classical” without using that word explicitly, certain architectural references reminiscent of Brunelleschi emerge, capturing geometric Renaissance perspectives. However, labeling a prompt “Renaissance painting” (not shown but performed in other side experiments) often triggers feminine portraits that do not obviously match canonical Renaissance aesthetics (excepted maybe Botticelli’s); Midjourney’s “classical paintings” also privilege idealized female beauty. Thus, AI seems to map *Renaissance* or *Classical* to specific themes rather than purely compositional elements.

*Viewing all images together.* After reviewing an entire batch of images side by side, the experts noted that their initial blind impressions occasionally shifted. For instance, aspects like *Clearness* might have been rated higher (e.g., near 4) had they seen the collective corpus from the start. In “A painting of a portrait” prompts, Midjourney often supplies female subjects regardless of “classical”, “neutral”, or “baroque”. If the label says “baroque”, the costuming or decorative details may indeed appear very Baroque (e.g., with typical and historically important ruffs or millstone collars), yet the composition stays rigidly centered—Classical in structure. Meanwhile, “classical” prompts display simpler, timeless clothing, and these subtleties are more evident when the images are grouped rather than intermixed randomly.

*Long descriptions and dynamic/static prompts.* When we used extended textual descriptions for the Classical style, DALL•E frequently produced coherent Renaissance-like architecture, channeling Brunelleschi’s experimentation and “windows onto the landscape”. This is interesting because the window was a central device in theoretical reflections on painting during the Renaissance; it was the instrument that enabled the development of perspective, a foundational shift in artistic representation. The Baroque prompts (in both DALL•E and Midjourney) leaned partly abstract yet still hinted at Baroque motifs. Interestingly, Midjourney “long description Baroque” often yielded purely abstract forms, whereas simply saying “baroque” triggered more historically recognizable details. In the “dynamic vs. static” scenarios, DALL•E sometimes resembled Baroque in lighting and clutter yet remained Classical in center-weighted composition; Midjourney produced similar visuals across both prompts. Importantly, prompting "dynamic painting" seems to produce images that are more chaotic, but a close inspection shows that this apparent chaos is most often organized around the center of the image, which confers it a somewhat Classical touch. Notably, *portraits* labeled “dynamic” vs. “static” in DALL•E had more pronounced differences (e.g., color palette, angles of the head), while Midjourney again displayed minimal variation across the two conditions. This holistic review thus reinforced the interplay of prompt wording, model biases, and the tension between compositional form (often Classical) and thematic or stylistic details (sometimes read as Baroque or dynamic).

As a final word, let us add that, as somehow observed in [33], this study highlights how Wölfflin’s stylistic concepts can arise in AI-generated outputs, yet remain uneven or incomplete relative to canonical art historical norms. It also underlines the fact that Wölfflin categories might have become outdated to analyze recent artistic productions, and that renewed, finer classes might need to be elaborated to provide more moderns analyzing keys to art historians.

### 4.7. Limitations and Future Works

*Reproducibility of the results.* By nature, the subjective expert-based evaluation stage cannot guarantee the full reproducibility of our results. To mitigate that issue, we used not only one but two experts in visual semiotics and art history (which are not easy to find), and asked them to review a large number of images in each experiment. This allows to reinforce the significance of our conclusions, seen as generic trends and observations rather than isolated scores. Regarding the generative stage alone, to the best of our knowledge, unfortunately neither DALL•E nor Midjourney allow fully reproducible results. This is due to a random pixel initialization at the start of the generation and to added randomness in the revision of the prompt and during the generative diffusion process itself. Just as for the evaluation, this also explains why we repeated each prompt multiple times. Therefore, even though the individual results (i.e., the images generated and their evaluations) are not fully reproducible, we established our experimental protocol to ensure as much as possible that our conclusions are significant, and hence that they are the parts benefiting from increased reproducibility.

*Choice of models.* While we focused on two models, DALL•E and Midjourney, our methodology can be used as is to assess any other text-to-image generative model, e.g., Stable Diffusion (https://stability.ai/stable-image (accessed on 18 February 2025)), Imagen (https://deepmind.google/technologies/imagen-3/ (accessed on 18 February 2025)), Flux (https://flux1.ai/) (accessed on 18 February 2025), etc. Our early tests with other models did not hint at drastically different behaviors than those observed with DALL•E and Midjourney. Hence, to keep the time-consuming and difficult expert-based evaluation manageable, we opted for those two popular and easy-to-use models. Also, we preferred not to generate all the images for all the models and then leave most of them unrated, as the expert evaluation is really what gives value to our experiments. Thus, as for the evaluation stage, the generation stage also ultimately focused on our two selected models. Comparing a wide range of models across a possibly restricted number of prompts would be an interesting future work.

*Extra prompting strategies.* We designed our prompting strategy with mostly short prompts in order to assess as much as possible how the models captured and materialized the essence of the concepts that we tested, alleviating any distraction. However, many other prompting strategies can be further tried. Experiment 3 is an example of a different kind of strategy, as it uses a highly structured and detailed prompt, yielding results on par with shorter prompts. We did not investigate other long prompts because their diversity explodes exponentially with the prompt length, making it more difficult to isolate influential factors and study specific aspects, such as each Wölfflin category. Yet, this is certainly an interesting idea for a follow-up work. Another prompting strategy to try could be few-shot prompting, where several iterations and refinements are allowed in order to maximize the chances of achieving a desired result. In our case, we used only one-shot prompting, as we believe this better reflects the core capabilities of the models, but it might well be the case that a small number of iterations yields different outcomes. Lastly, one could test image-conditioned prompting, where a reference image is passed along the prompt to the model, as done in [38] to mix various styles. This could serve to guide the model toward a more meaningful result than simply letting the model generate images out of nowhere, which in turn might not allow an insightful analysis. Again, in this paper, we chose to focus on the intrinsic properties of the models, but we believe that many prompting strategies could serve as basis for interesting future work.

*Multivariate analysis.* Although a multivariate approach could reveal deeper structures in how experts rate Wölfflin’s dimensions, we ultimately chose not to pursue this route. The core reason is that it would shift the focus of the analysis too much on the agreement between the experts or on very peculiar patterns, thus many more experts would be needed to fully deliver an insightful message. Moreover, layering additional complexity onto our already multifaceted experiments might risk obscuring the interpretive, art historical insights that are central to this study. More in-depth data science-based analysis would benefit from a more restricted and data rich experimental context, which could ultimately consist in a paper on its own, with its own research questions. Instead, descriptive analyses and expert commentaries allow us to preserve clarity and accessibility for our intended audience, ensuring that the theoretical narrative around Wölfflin’s framework remains at the forefront.

## 5. Conclusions

In this study, we examined how two popular text-to-image models (DALL•E and Midjourney) interpret and generate imagery under Heinrich Wölfflin’s Classical/Baroque framework. Through both explicit style prompts (e.g., “baroque”, “classical”) and more nuanced compositional instructions (“dynamic”, “static”, or reworked Wölfflin descriptors), we collected expert ratings under blind conditions, then followed up with a holistic review of the resulting images. Our results reveal a complex interplay between prompt wording, model biases, and expert interpretation. While “baroque” prompts often elicit dramatic contrasts or mixed figures, “classical” tends to be less distinctly defined, especially when the genre (e.g., portrait, still life) imposes a centered composition. In some cases, “dynamic” versus “static” triggers equally large stylistic ratings, showing how neither label is guaranteed to produce the expected historical cues. The ”dynamic image” prompt even shows seemingly chaotic images which, in fact, display a Classical spatial organization around their center. At times, Baroque themes appear within fundamentally Classical compositions, indicating a tension between the two styles. Meanwhile, removing explicit style labels but retaining Wölfflin-based descriptions yields more abstract forms, detached from historic Baroque figurations. The broader, side-by-side review helped the experts identify subtleties—like the partial overlap in clearness or lighting—that were not immediately apparent when images were rated in isolation. Taken together, these findings illustrate that while Wölfflin’s categories can influence AI outputs, the models’ contemporary training data and generative preferences complicate any straightforward mapping of Classical versus Baroque. Our work thus suggests that Wölfflin’s theory, once deemed atemporal, merits thoughtful revision to address the novel ways generative systems blend compositional form, thematic cues, and modern biases.

## Figures and Tables

**Table 1:** Experiment 1 average Likert scores from two experts (E1 and E2) per model (DAL stands for DALL•E, MidJ for Midjourney) and per prompt “*A classical painting*”, “*A painting*” (neutral), “*A baroque painting*”. Column (1) represents the experts’ general feeling about the Classical vs. neutral vs. Baroque aspect of the images, and columns (2)–(6) correspond to Wölfflin’s categories as follows: (2) Linear vs. Painterly; (3) Planar vs. Recessional; (4) Closed vs. Open Form; (5) Multiplicity vs. Unity; and (6) Clearness vs. Unclearness. Scores range from 1 (strongly Classical) to 5 (strongly Baroque).

Condition	Expert 1 (E1)						Expert 2 (E2)					
	(1)	(2)	(3)	(4)	(5)	(6)	(1)	(2)	(3)	(4)	(5)	(6)
**DAL Classical**	1.8	1.7	2.5	2.4	2.5	2.7	2.0	2.1	2.1	2.2	2.0	2.3
**DAL Neutral**	2.0	1.7	2.5	2.5	2.3	2.7	1.6	1.8	1.4	2.0	1.8	2.6
**DAL Baroque**	4.1	3.3	3.6	3.3	3.8	4.4	3.2	3.2	3.0	3.0	3.2	3.6
**MidJ Classical**	2.0	1.8	2.0	2.3	2.4	2.7	3.5	3.8	3.7	3.3	3.2	3.3
**MidJ Neutral**	3.2	2.7	3.1	3.3	3.3	3.7	3.8	3.7	3.5	3.3	4.0	3.8
**MidJ Baroque**	4.2	3.0	4.1	3.8	4.0	4.1	3.9	4.0	3.8	3.4	3.7	3.7

**Table 2:** Experiment 2 average Likert scores from two experts (E1 and E2) per model (DAL stands for DALL•E, MidJ for Midjourney) and per prompt—“*A classical painting of a portrait*”, “*A painting of a portrait*” (neutral), “*A baroque painting of a portrait*”. Column (1) represents the experts’ general feeling about the Classical vs. neutral vs. Baroque aspect of the images, and columns (2)–(6) correspond to Wölfflin’s categories: (2) Linear vs. Painterly; (3) Planar vs. Recessional; (4) Closed vs. Open Form; (5) Multiplicity vs. Unity; and (6) Clearness vs. Unclearness. Scores range from 1 (strongly Classical) to 5 (strongly Baroque).

Condition	Expert 1 (E1)						Expert 2 (E2)					
	(1)	(2)	(3)	(4)	(5)	(6)	(1)	(2)	(3)	(4)	(5)	(6)
**DAL Classical**	2.2	2.3	1.9	2.1	2.5	3.4	3.0	3.1	2.8	2.7	2.7	3.4
**DAL Neutral**	2.2	2.0	1.7	1.9	2.0	2.9	2.9	3.1	2.6	2.7	2.9	2.7
**DAL Baroque**	3.0	2.5	2.7	2.8	2.7	3.5	3.2	3.2	3.1	2.8	2.9	3.5
**MidJ Classical**	2.2	1.7	1.7	2.1	2.1	2.8	3.7	4.0	3.1	3.2	3.0	3.7
**MidJ Neutral**	2.5	2.5	1.8	2.2	2.1	2.5	3.8	3.8	3.5	3.2	3.4	3.6
**MidJ Baroque**	2.1	1.9	1.8	2.5	2.4	3.3	2.9	3.3	2.7	2.8	2.9	3.4

**Table 3:** Experiment 3 average Likert scores from two experts (E1 and E2) per model (DAL stands for DALL•E, MidJ for Midjourney) and per prompt using a long description of Classical and Baroque characteristics (see text for details). Column (1) represents the experts’ general feeling about the Classical vs. neutral vs. Baroque aspect of the images, and columns (2)–(6) correspond to Wölfflin’s categories: (2) Linear vs. Painterly; (3) Planar vs. Recessional; (4) Closed vs. Open Form; (5) Multiplicity vs. Unity; and (6) Clearness vs. Unclearness. Scores range from 1 (strongly Classical) to 5 (strongly Baroque).

Condition	Expert 1 (E1)						Expert 2 (E2)					
	(1)	(2)	(3)	(4)	(5)	(6)	(1)	(2)	(3)	(4)	(5)	(6)
**DAL Classical**	1.4	1.1	2.0	2.2	1.6	2.1	3.3	2.6	3.3	3.5	3.1	2.3
**DAL Baroque**	3.3	2.8	3.3	2.8	2.8	3.9	3.6	3.7	3.5	3.5	3.7	3.1
**MidJ Classical**	2.1	1.5	2.4	2.0	1.7	3.0	2.9	3.0	2.9	2.8	2.8	3.4
**MidJ Baroque**	3.9	3.2	3.8	3.6	3.1	4.0	4.0	3.9	3.9	4.0	4.0	3.7

**Table 4:** Experiment 4 average Likert scores from two experts (E1 and E2) per model (DAL stands for DALL•E, MidJ for Midjourney) and per prompt—“*A dynamic painting*” and “*A static painting*”. Column (1) represents the experts’ general feeling about the dynamic vs. static aspect of the images, and columns (2)–(6) correspond to Wölfflin’s categories: (2) Linear vs. Painterly; (3) Planar vs. Recessional; (4) Closed vs. Open Form; (5) Multiplicity vs. Unity; and (6) Clearness vs. Unclearness. Scores range from 1 (strongly static) to 5 (strongly dynamic).

Condition	Expert 1 (E1)						Expert 2 (E2)					
	(1)	(2)	(3)	(4)	(5)	(6)	(1)	(2)	(3)	(4)	(5)	(6)
**DAL Static**	1.9	1.7	2.0	2.3	1.9	2.4	2.2	2.2	1.8	2.2	2.1	2.7
**DAL Dynamic**	2.7	2.2	3.3	3.4	2.8	2.1	3.6	3.4	3.5	3.4	3.3	2.2
**MidJ Static**	3.2	2.9	3.0	2.9	3.1	2.5	4.0	4.0	4.0	3.8	3.6	2.9
**MidJ Dynamic**	3.2	2.9	3.2	3.4	3.2	2.5	4.0	4.0	3.9	3.8	4.0	2.7

**Table 5:** Experiment 5 average Likert scores from two experts (E1 and E2) per model (DAL stands for DALL•E, MidJ for Midjourney) and per prompt—“*A dynamic painting of a portrait*” and “*A static painting of a portrait*”. Column (1) represents the experts’ general feeling about the dynamic vs. static aspect of the images, and columns (2)–(6) correspond to Wölfflin’s categories: (2) Linear vs. Painterly; (3) Planar vs. Recessional; (4) Closed vs. Open Form; (5) Multiplicity vs. Unity; and (6) Clearness vs. Unclearness. Scores range from 1 (strongly static) to 5 (strongly dynamic).

Condition	Expert 1 (E1)						Expert 2 (E2)					
	(1)	(2)	(3)	(4)	(5)	(6)	(1)	(2)	(3)	(4)	(5)	(6)
**DAL Static**	1.7	1.6	2.1	2.0	1.9	2.7	1.9	2.1	1.8	1.8	1.9	2.9
**DAL Dynamic**	2.6	2.4	3.0	3.1	2.5	3.5	2.9	3.2	3.0	2.4	2.9	2.8
**MidJ Static**	2.4	2.6	2.6	2.2	2.1	2.9	3.8	4.0	4.0	3.1	3.4	3.3
**MidJ Dynamic**	3.1	2.8	2.6	2.4	2.4	3.2	3.9	4.0	4.0	3.1	3.5	2.7

**Figure A1:**
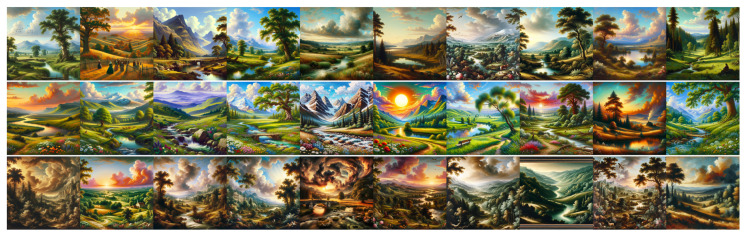
Images produced by DALL•E for Experiment 2. **Top line**: “*A classical painting of a landscape*”; **middle line**: “*A painting of a landscape*” (neutral); and **bottom line**: “*A baroque painting of a landscape*”.

**Figure A2:**
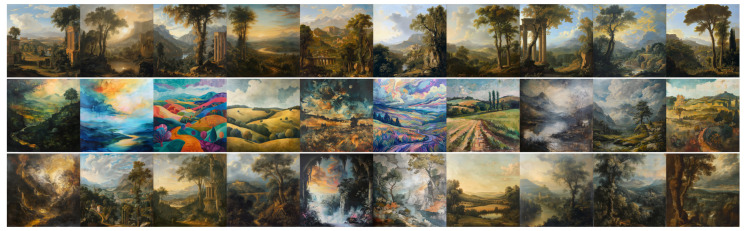
Images produced by Midjourney for Experiment 2. **Top line**: “*A classical painting of a landscape*”; **middle line**: “*A painting of a landscape*” (neutral); and **bottom line**: “*A baroque painting of a landscape*”.

**Figure A3:**
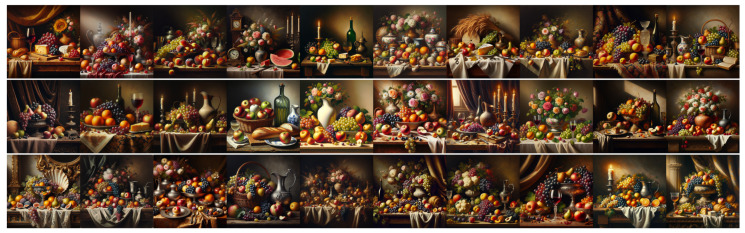
Images produced by DALL•E for Experiment 2. **Top line**: “*A classical painting of a still life*”; **middle line**: “*A painting of a still life*” (neutral); and **bottom line**: “*A baroque painting of a still life*”.

**Figure A4:**
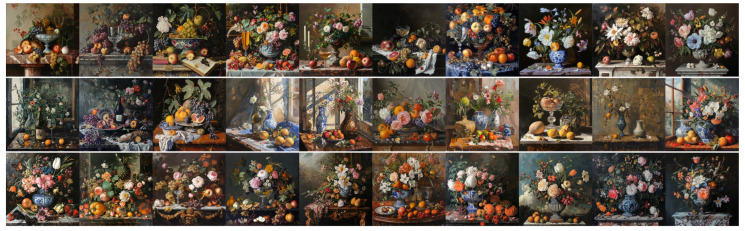
Images produced by Midjourney for Experiment 2. **Top line**: “*A classical painting of a still life*”; **middle line**: “*A painting of a still life*” (neutral); and **bottom line**: “*A baroque painting of a still life*”.

## Data Availability

All images generated and expert ratings will be made available upon request.

## Appendix A

This appendix provides the images produced but not rated by our experts to study the impact of the genre (with “landscape” and “still life”) on the Classical vs. Baroque orientation of the models, as done with "portraits" in Section 4.2. The formal rating was omitted because, by quickly looking at them, our experts said that the same observations as for portraits apply, hence yielding redundant information.
